# WD40-Repeat Proteins in Plant Cell Wall Formation: Current Evidence and Research Prospects

**DOI:** 10.3389/fpls.2015.01112

**Published:** 2015-12-22

**Authors:** Gea Guerriero, Jean-Francois Hausman, Inés Ezcurra

**Affiliations:** ^1^Environmental Research and Innovation, Luxembourg Institute of Science and TechnologyEsch-sur-Alzette, Luxembourg; ^2^School of Biotechnology, Division of Industrial Biotechnology, KTH Royal Institute of Technology, AlbaNova University CenterStockholm, Sweden

**Keywords:** WDR proteins, protein–protein interaction, plant cell wall, genomic collinearity, lignocellulose

## Abstract

The metabolic complexity of living organisms relies on supramolecular protein structures which ensure vital processes, such as signal transduction, transcription, translation and cell wall synthesis. In eukaryotes WD40-repeat (WDR) proteins often function as molecular “hubs” mediating supramolecular interactions. WDR proteins may display a variety of interacting partners and participate in the assembly of complexes involved in distinct cellular functions. In plants, the formation of lignocellulosic biomass involves extensive synthesis of cell wall polysaccharides, a process that requires the assembly of large transmembrane enzyme complexes, intensive vesicle trafficking, interactions with the cytoskeleton, and coordinated gene expression. Because of their function as supramolecular hubs, WDR proteins could participate in each or any of these steps, although to date only few WDR proteins have been linked to the cell wall by experimental evidence. Nevertheless, several potential cell wall-related WDR proteins were recently identified using *in silico* approaches, such as analyses of co-expression, interactome and conserved gene neighborhood. Notably, some WDR genes are frequently genomic neighbors of genes coding for GT2-family polysaccharide synthases in eukaryotes, and this WDR-GT2 collinear microsynteny is detected in diverse taxa. In angiosperms, two WDR genes are collinear to cellulose synthase genes, *CesA*s, whereas in ascomycetous fungi several WDR genes are adjacent to chitin synthase genes, *chs*. In this Perspective we summarize and discuss experimental and *in silico* studies on the possible involvement of WDR proteins in plant cell wall formation. The prospects of biotechnological engineering for enhanced biomass production are discussed.

## Plant Cell Wall Biosynthesis: A Multiplayer Matter

Plant lignocellulosic biomass is one of the most abundant renewable resources on Earth, an important feedstock for the supply of polymers and chemicals and an alternative to the use of petroleum (Guerriero et al., 2014a, 2015a). It is mainly composed of secondary cell walls, heterogeneous composites of polysaccharides (cellulose, hemicelluloses) and proteins impregnated with the aromatic polymer lignin, which confers mechanical support and recalcitrance to degradation (Guerriero et al., 2015b). Cell walls are dynamic structures which are continuously synthesized and remodeled during plant development to accommodate the needs of the growing plant cells and the broad variety of cell shapes (Guerriero et al., 2014b). The biosynthesis of plant cell walls is a complex and highly regulated process and several aspects have been thoroughly investigated. This has been possible thanks to the large amount of data generated via transcriptomics, proteomics, metabolomics on both woody and herbaceous species (Lin et al., 2013; Printz et al., 2015) and to functional studies in model plants, i.e., *Arabidopsis thaliana* (Anderson et al., 2015). The increasing number of sequenced plant genomes and genome-wide analyses of cellulose and lignin-related genes (Peng et al., 2013; Myburg et al., 2014) has further contributed to our understanding of plant cell wall biosynthesis. Moreover, the availability of plant cell culture systems differentiating tracheary elements (Oda et al., 2005) has provided a valuable tool to study the sequential steps of secondary cell wall biosynthesis using high-resolution imaging techniques (Lacayo et al., 2010).

Plant cell wall biosynthesis is regulated during different stages of gene expression, namely at the transcriptional and post-translational level (Hijazi et al., 2014; Zhong and Ye, 2014). A transcriptional wiring composed of master and downstream regulators determines the regulation of cell wall structural genes, typically encoding cellulose synthases and other carbohydrate-active enzymes, or enzymes in lignin biosynthesis. This hierarchical organization is conserved across different species, from woody to herbaceous, from monocots to dicots (Winzell et al., 2010; Zhao and Bartley, 2014).

At the post-translational level, plant wall biosynthesis requires the assembly of large protein complexes at the membranes, a process which relies upon intensive intracellular trafficking (Wightman and Turner, 2010) and cytoskeleton interactions (Gutierrez et al., 2009). A key example entails the cellulose synthase complex (CSC, a.k.a the “rosette terminal complex”) which is pre-assembled in the Golgi and then delivered in vesicles to the plasma membrane (Wightman and Turner, 2010). The complex was recently shown to be composed of six particles, each containing a trimer of cellulose synthase catalytic subunits (CESA4, CESA7, CESA8) in equimolar stoichiometry (1:1:1), in *A. thaliana* secondary cell walls (Hill et al., 2014). CSC establishes interactions with other proteins during its vesicle trafficking to and from the membrane (Gutierrez et al., 2009) and at the plasma membrane (Vain et al., 2014). Protein–protein interactions are likewise crucial for the biosynthesis of non-cellulosic polysaccharides in the Golgi (reviewed by Oikawa et al., 2013) and for the organization of multienzyme complexes, or “metabolons” (Jørgensen et al., 2005), which maximize shunting of metabolites into branches of the plant secondary metabolism that are relevant to the cell wall, such as the phenylpropanoid pathway (Chen et al., 2014).

It is therefore clear that the formation of supramolecular protein complexes is crucial for cell wall biosynthesis in plants, and in this perspective we aim at understanding more about the scaffolding components mediating protein–protein interaction, to inspire additional biotechnological strategies to tailor plant cell wall biosynthesis. We focus on a widely distributed family of scaffolding proteins, the WD40-repeat proteins (WDRs), and discuss their role in cell wall biosynthesis by presenting evidence linking these proteins to cell wall-related processes. We also present potential biotechnological uses of plant WDRs to modulate lignocellulose synthesis.

## Plant WDR Proteins Have Pleiotropic Functions

**FIGURE 1:**
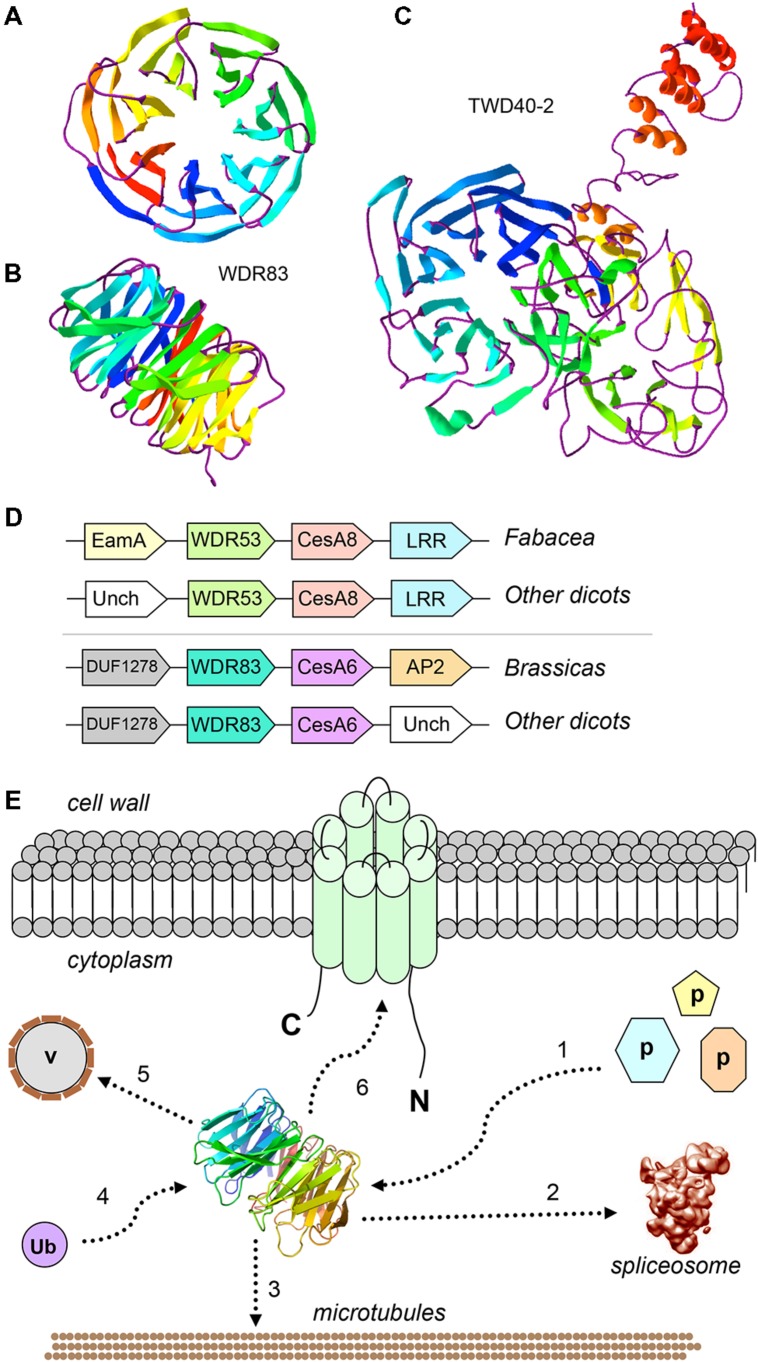
**Cell wall-related WDRs: predicted 3D structure, conserved genomic neighbors of *CesA*s, and model of their possible roles.** **(A)** Predicted 3D structure of AtWDR83 (AT5G64730), view from the top of the propeller, showing the seven blades; **(B)** view from the side, showing the toroidal structure with top, bottom and circumferential sites of interactions; **(C)** View of predicted 3D structure of TWD40-2 (AT5G24710), showing the two seven-bladed propellers; The models were obtained with Phyre2 (Kelley et al., 2015; available at http://www.sbg.bio.ic.ac.uk/phyre2/html/page.cgi?id=index) and visualized with Swiss-PdbViewer (Guex and Peitsch, 1997; available at http://www.expasy.org/spdbv/). Templates for the AtWDR83 model were multiple WDR proteins, including Gbeta5/RGS9, ribosomal Rsa4, and pre-mRNA-splicing factor prp5, whereas the TWD40-2 template was the coatomer subunit alpha, alpha-COP; **(D)** Conserved microcolinearity of *WDR* and *CesA* genes in angiosperms. Unch, Uncharacterized; EamA, EamA-like transporter; LRR, Leucine-rich repeat; **(E)** Hypothetical model showing the possible functions of WDR53/WDR83-like proteins in cellulose biosynthesis. 1: WDR53/WDR83 represent a “platform” for the binding of unknown cell wall-related proteins; 2: WDR53/WDR83 interact with the spliceosome and splices *CesA* transcripts; 3: WDR53/WDR83 interact with the cytoskeleton; 4: WDR53/WDR83 bind ubiquitin; 5: WDR53/WDR83 mediate trafficking of Golgi vesicles (v); 6: WDR53/WDR83 interact with CESA complexes at the plasma membrane.

Plants are among the most complicated organisms to study from a systems biology perspective. Their metabolic redundancy, a consequence of their sessile lifestyle (Mishra et al., 2012), determines intricate interactome maps (Morsy et al., 2008). Protein–protein interactions coordinate the formation of supramolecular complexes which ensure the correct execution of sequential steps in a specific metabolic pathway. The association of proteins, either temporal or stable, requires usually the presence of ancillary scaffolding proteins. In eukaryotes a family of scaffolding proteins, the WDR proteins, participates in assembling protein complexes (Stirnimann et al., 2010). The structure of these proteins is a β-propeller, where several repeating units, composed of ca 40–60 amino acids (among which conserved GH and WD residues), fold into four-stranded anti-parallel β-sheets (Stirnimann et al., 2010; Mishra et al., 2012). The β-sheets arrange circularly around a central axis, thereby creating a multi-bladed toroidal structure which can establish molecular interactions via the top, bottom, and circumference (**Figures 1A,B**). WDR proteins are rigid platforms that can interact with many partners, a phenomenon known as “moonlighting.” This promiscuity explains the wide range of cellular processes in which they are implicated (Stirnimann et al., 2010; Mishra et al., 2012), which span from (a)biotic stress response to intracellular trafficking and transcriptional regulation, just to name a few.

## Plant WDR Proteins with Roles in Cell Wall-Related Processes

Given their functional versatility and scaffolding properties, WDR proteins are plausible candidates for roles in cell wall biosynthesis, although to date only three WDR proteins have been associated to the cell wall through experimental evidence, namely FRAGILE FIBER3 (FRA3), LEUNIG Homolog (LUH) and TWD40-2. In *Arabidopsis*, FRA3 is a type II inositol polyphosphate 5-phosphatases containing an N-terminal WDR domain (Zhong et al., 2004). Mutations in the *FRA3* gene cause phenotypes related to a reduced secondary cell wall thickness in fibers and xylem vessels, with consequent decrease in stem strength (Zhong et al., 2004). Analysis of cell wall composition showed that the mutants have a 37% reduction in glucose, thereby suggesting that cellulose is mainly affected by the mutations (Zhong et al., 2004). Moreover, the actin organization in fiber cells is compromised with consequences on trafficking of vesicles containing CESA complexes and/or cell wall polysaccharides. The second *Arabidopsis* WDR protein, LUH, is a transcriptional co-repressor belonging to the Gro/TLE family (Bui et al., 2011). *Arabidopsis luh-1* mutants have defects associated with mucilage extrusion, which are reminiscent of the *mum2* mutants (*mucilage modified 2*, a β-galactosidase involved in mucilage maturation). LUH and (possibly) the closely related WDR protein LEUNIG (LUG) regulate the expression of cell wall-related genes associated with mucilage maturation (Bui et al., 2011). The penetrance of the *luh-1* phenotype indicates that this WDR protein is a key regulator of cell wall modifications associated with a critical phase of seed coat maturation (Bui et al., 2011). The third *Arabidopsis* WDR protein, TWD40-2, was recently linked to clathrin-mediated endocytosis of cellulose synthases during cellulose biosynthesis (Bashline et al., 2015). Mutation of the *TWD40-2* gene caused impaired endocytosis, unregulated overaccumulation of CESAs at the plasma membrane, decreased cellulose content and stunted plant growth. This highlights the importance of controlled CESA endocytosis in regulating cellulose biosynthesis, and the TWD40-2 WDR protein is key in this process. TWD40-2 is unique to plants and some protozoan species, and is similar to the alpha and beta coatomer WDR subunits, alpha- and beta-COP (Zhang et al., 2015). Although TWD40-2 is structurally related to alpha- and beta-COP, with its two N-terminal propellers and a C-terminal alpha-helical region (**Figure 1C**), it is unclear whether it coats endocytic vesicles as alpha- and beta-COP do (Dodonova et al., 2015).

## WDR Proteins Linked to the Cell Wall by *In Silico* Predictions

Although only three WDR proteins have been linked to the cell wall by biochemical and/or genetic evidence, several potential cell wall-related WDR proteins were recently identified using *in silico* approaches, such as analyses of co-expression, interactome and conserved gene neighborhood. A WDR gene (At5g53500) was found to be highly co-regulated with the primary cell wall *CesA1*, *CesA3*, and *CesA6* by regression analysis of public microarray data sets (Persson et al., 2005). More recently, a computational framework was developed for the prediction of proteins related to cell wall synthesis, based on known cell wall synthesis-related proteins and their predicted protein-protein interactions in the *Arabidopsis* interactome, which is inferred from multiple indirect lines of evidence, including coexpression, colocalization, coevolution, annotation similarity, domain interaction, and homologous interactions in other species (Geisler-Lee et al., 2007; Zhou et al., 2010). The study identified three WDR proteins, AT1G04510, AT2G43770, and AT4G34460, among the 42 most likely cell wall-related candidate proteins. Other studies show that AT1G04510 codes for MAC3A, a WDR protein with roles in plant immunity and activation of the spliceosome (Monaghan et al., 2009). The computational analysis predicts that MAC3A/AT1G04510 interacts with a rhamnose synthase, RHM1 (AT1G78570), involved in accumulation of the pectic polysaccharide rhamnogalacturonan in the cell wall. Both functions of MAC3A, spliceosome activation and plant defense, are consistent with a possible role in the cell wall, as both processes may entail the cell wall (Huang et al., 2013; Bellincampi et al., 2014). Further, cell wall pectin is an important factor in plant defense (Bellincampi et al., 2014), and thus the predicted interaction of MAC3A/AT1G04510 with RHM1 is in agreement with its function. Then, MAC3A/AT1G04510 could potentially regulate plant defense through splicing of the RHM1/AT1G78570 transcript, in analogy to regulation by splicing of Callose Synthase5 (Huang et al., 2013), although this remains to be shown experimentally. The second predicted cell wall-related WDR protein, AT2G43770, is the plant homolog of SNRNP40, also a spliceosome component, and is predicted to interact with a UDP-D-xylose synthase, UXS6 (AT2G28760). Finally, the third predicted cell wall WDR protein, AT4G34460, is AGB1, a G-Protein β subunit, and the study predicts its interaction with a GDP-D-mannose 3′,5′-epimerase (AT5G28840). The role of AGB1 in the cell wall was experimentally confirmed in later studies showing its involvement in cell wall defense (Delgado-Cerezo et al., 2012), suggesting a fair level of accurate predictions of cell wall-involved WDRs by the computational framework.

## Conserved Collinear Microsynteny between WDR and Glycan Synthase Genes in Diverse Eukaryote Taxa

The clustering of functionally related genes is prevalent in prokaryotes but was conventionally assumed to be less common in eukaryotes. However, this notion is shifting as clusters of functionally related genes are increasingly detected in eukaryotes (Hurst et al., 2004; Yi et al., 2007) including, recently, plants (Nützmann and Osbourn, 2014). Although gene clusters in plant and fungi concern secondary metabolism, a recent bioinformatic study identified a cell wall gene cluster in fungi (Pacheco-Arjona and Ramirez-Prado, 2014), suggesting that conserved genomic neighborhood may provide a means to identifying genes involved in processes other than secondary metabolism. The function of physically clustering functionally related genes in eukaryotes is unclear, but could involve coordinated gene expression or keeping genes together to avoid toxic effects of their individual loss.

We previously identified two cases of conserved genomic collinearity between a WDR gene and a *CesA* in the genomes of several dicots (Guerriero et al., 2012). Specifically, a plant *WDR53* gene (represented by At5g45760 in *Arabidopsis*) is collinear with the secondary cell wall *CesA8*, while a *WDR83* (At5g64730) is adjacent to the primary cell wall *CesA6* (schematized in **Figure 1D**). We showed that *WDR53* and *CesA8* are partially co-expressed in apple and *Populus* (Guerriero et al., 2012). Further, we identified a bicistronic *WDR53-CesA8* transcript in phytoplasma-infected apple tissues, where the *CesA8* cistron is alternatively spliced and lacks a portion corresponding the RING-type zinc finger domain. We speculate on whether this alternatively spliced *CesA8* cistron is translated in order to alter cellulose biosynthesis in response to phytoplasma infection.

We have also showed that the genomic association between a WDR gene and a gene encoding a glycosyltransferase from family 2 (GT2) is a conserved feature in eukaryotes, since, besides dicots, it is reported in metazoans (hyaluronan synthase-WDR microsynteny) and in fungi (chitin synthase-WDR microsynteny; Guerriero et al., 2012). In fungi, the genomic collinearity between a chitin synthase (*chs*) and a WDR gene is conserved among several species (Guerriero et al., in revision). This WDR gene (AN10216 in *Aspergillus nidulans*) occurs mainly in fungi and plants, and is therefore named Fungal-Plant WDR (*FPWD*). *FPWD* is localized within a recently identified cell wall metabolism gene cluster involving among others, besides *chsD*, a Chs activator, a myosin V, a GH17 cell wall glucanase, scw11 (Pacheco-Arjona and Ramirez-Prado, 2014). Deletion of *FPWD* and a tightly linked neighboring beta-flanking gene causes cell wall and chitin-related phenotypes in *A. nidulans*, confirming *FPWD*’s role in the cell wall (Guerriero et al., in revision). These results suggest that identifying conserved gene neighbors may be a useful bioinformatic approach to mining for gene targets, in addition to more traditional approaches based on gene expression, proteomics or interactome inference.

In light of the conserved genomic microsynteny *WDR53-CesA8* and *WDR83-CesA6*, it is then plausible to assume a cellulose biosynthesis-related role for these WD40 proteins. Given the moonlighting properties of many plant WDR proteins, they could be involved in one or several processes involving the delivery of the CSC to membrane microdomains, e.g., via regulating transcript splicing, vesicle trafficking, the formation of platforms mediating protein-protein interactions and/or protein turnover via ubiquitination (schematized in **Figure 1E**).

In flowering plants, the *FPWD* gene occurs as a small gene family of around five genes of unknown function, represented in *Arabidopsis* by At1g78070, At1g36070, At1g55680, At3g13340, and At5g56190. We noticed that the plant *FPWD*s are conserved genomic neighbors of cell wall or carbohydrate-related genes, coding for trehalose-6-phosphate phosphatase, GH3 and actin ACT7 (in higher plants), as well as nucleotide sugar transporter and GT57 glucosyltransferase (in green algae). Examination of their expression using publicly available microarray databases in *Arabidopsis* and *Populus* suggests that they may be either largely xylem-specific, or pollen-specific (data not shown), consistent with a possible role in the cell wall. Clearly, the precise functions of these potentially wall-involved WDRs, and of their gene neighbors, remain to be further elucidated.

## Phylogenetic Relationships of Cell Wall Candidate WDR Proteins

We here identify a number of plant WDR proteins with confirmed or potential roles in the lignocellulosic cell wall. A recent phylogenetic analysis of all *Arabidopsis* (comprising 237 gene ids) and rice (comprising 200 gene ids) WDRs established that the plant WDR phylogenetic tree consists of five major branches, numbered I–V (Ouyang et al., 2012). To better understand the function and evolution of the cell wall candidate WDRs, we analyzed their cluster location within the phylogenetic tree (**Table 1**). We observe that cell wall-related WDR proteins belong to distinct phylogenetic clusters: WDR83, MAC3A, AT2G43770 and AGB1 belong to cluster I, together with WDR5, involved in histone methylation (Jiang et al., 2011). WDR53 is in cluster III with FRA3, involved in secondary cell wall formation, ROOT INITIATION DEFECTIVE 3, RID3, with roles in pre-rRNA processing (Shinohara et al., 2014), and SPIRRIG, a BEACH domain WDR which regulates mRNA stability (Steffens et al., 2015). Further, the FPWDs branch in cluster II, together with the TOPLESS-related, TPR, protein cluster, which are involved in chromatin-mediated repression by histone deacetylation (Pi et al., 2015), and RBBP5 LIKE, RBL, involved in histone methylation (Jiang et al., 2011). Finally, TWD40-2, involved in clathrin mediated endocytosis of CESA complexes, also branches in cluster II. In summary, the cell wall candidate WDR proteins do not cluster together but are rather widely distributed across the plant WDR superfamily.

**Table 1:** Phylogenetic cluster location of cell wall-related WDR proteins (upper section), listed together with representative members of each cluster (lower section).

Gene ID	Annotation	Phylo-genetic cluster	Function
AT1G04510	MAC3A	I	Splicing, plant defense
AT2G43770	–	I	Unknown
AT4G34460	AGB1	I	Cell wall defense
AT5G64730	WDR83	I	Unknown
AT1G78070	FPWD-1	II	Unknown
AT1G36070	FPWD-2	II	Unknown
AT1G55680	FPWD-3	II	Unknown
AT3G13340	FPWD-4	II	Unknown
AT5G56190	FPWD-5	II	Unknown
AT5G24710	TWD40-2	II	Cell wall
AT1G65580	FRA3	III	Cell wall
AT3G50590	TWD40-1	III	Cell wall
AT5G45760	WDR53	III	Unknown

AT3G49660	WDR5	I	Histone methylation
AT5G27030	TPR3	II	Chromatin regulation
AT3G21060	RBL	II	Histone methylation
AT3G49180	RID3	III	Pre-rRNA processing
AT1G03060	SPIRRIG, SPI	III	Membrane; RNA processing


## Biotechnological Prospects: WDR Proteins to Boost Plant Biomass Production

The depletion of fossil fuel pushes toward the identification and use of renewable energy sources to promote the development of a circular economy. In this perspective plant biomass is very important, since it constitutes one of the most abundant sustainable resources for humanity and it can provide biopolymers, fibers, biofuel, thereby promoting economic growth, while minimizing environmental impacts. Besides woody species like *Populus* and *Eucalyptus*, fast growing herbaceous species, like the fiber-crops flax and hemp, have also considerable importance for the bio-economy, because of their shorter growth cycles, their high biomass yield (e.g., 24 tons/hectare for hemp; Kolaříková et al., 2013) and the presence of cellulosic fibers in their stems (the so-called bast fibers; Guerriero et al., 2013). Because of these advantageous characteristics and the properties of their fibers (length, strength, renewability), these crops are widely used in industry to provide fibers for the reinforcement of composites. For the bio-economy high biomass-production is a sought-after character and biotechnological strategies aimed at boosting biomass formation are attracting research efforts (Nieminen et al., 2012; Dubouzet et al., 2013). Here we identify a list of WDR proteins with confirmed or potential roles in the lignocellulosic cell wall, which are interesting targets for engineering plants with enhanced biomass production. Such effort would involve the use of promoters to drive altered expression in the cambium or xylem (Ji et al., 2010; Ratke et al., 2015), to influence xylem proliferation and/or lignocellulose biosynthesis. As one possible example, seedlings of *twd40-2* mutants display reduced endocytosis, cellulose content, and plant growth. The *TWD40-2* gene is expressed in most tissues, but its expression is highest in stem or xylem in *Arabidopsis* and *Populus* according to the BAR eFT gene expression browser (http://bar.utoronto.ca/; data not shown). Then, enhancing *TWD40-2* expression in the xylem could increase cellulose content. A further approach to enhance the function of target WDR proteins could potentially involve mutation of selected surface residues involved in protein interaction, a.k.a. “interaction hotspots,” in order to decrease the target’s eventual pleiotropy. This would require identification of interaction hotspots for example using tools for *in silico* prediction, such as modeling their 3D structure, combined with experimental confirmation. The ultimate goal of such mutagenesis, which could be generated through genome editing, would be to reduce pleiotropy or moonlighting of the target WDR, and obtain more specialized enzymes, to avoid undesired side effects.

## Author Contributions

GG and IE conceived the idea of writing the Perspective paper. GG and IE obtained the bioinformatic data. GG, J-FH, and IE interpreted the data and wrote the manuscript.

## Conflict of Interest Statement

The authors declare that the research was conducted in the absence of any commercial or financial relationships that could be construed as a potential conflict of interest.
